# Reliability of pre-resection ligament tension assessment in imageless robotic assisted total knee replacement

**DOI:** 10.1186/s42836-024-00266-y

**Published:** 2024-09-02

**Authors:** Dennis K. H. Yee, Jonathan T. C. Leung, Vikki Chu, Gene Man, Gloria Y. T. Lam, Jimmy K. Y. Lau, Tsz-Lung Choi, Wai-Wang Chau, Jonathan Patrick Ng, Michael Tim-Yun Ong, Kevin Ki-Wai Ho, Patrick Shu-Hang Yung

**Affiliations:** 1 Department of Orthopaedics & Traumatology, Alice Ho Miu Ling Nethersole Hospital, Hong Kong SAR, China; 2 Office of Research and Knowledge Transfer Services, Chinese University of Hong Kong, Hong Kong SAR, China; 3 Department of Orthopaedics and Traumatology, Chinese University of Hong Kong, Hong Kong SAR, China; 4 Department of Orthopaedics and Traumatology, Prince of Wales Hospital, Hong Kong SAR, China; 5 Department of Orthopaedics and Traumatology, CUHK Medical Centre, Hong Kong SAR, China

**Keywords:** Ligament tension assessment, Pre-excision, Reliability analysis, Robotic-assisted surgery, Imageless, Total knee replacement

## Abstract

**Background:**

Ligament tension balance is a major determinant for the success of total knee replacement (TKR). The present study aimed at determining the inter-rater and intra-rater reliability in performing ligament tension assessment using an imageless robotic-assisted TKR.

**Methods:**

Twenty-four knees in 21 patients who received robotic-assisted TKR for end-stage varus osteoarthritis were examined. Three orthopedic specialists and six orthopedic trainees participated in the operations. Data from the ligament tension assessment were collected during the operations.

**Results:**

For the inter-rater reliability, “extension medial” and “flexion medial” had excellent reliability whilst “extension lateral” and “flexion lateral” had good-to-excellent reliability. For the intra-rater reliability, “extension medial” showed excellent reliability, “extension lateral” and “flexion medial” showed good-to-excellent reliability, and “flexion lateral” showed moderate-to-excellent reliability.

**Conclusions:**

Robotic-assisted technology provides a reliable solution to improve ligament tension assessment. All ligament tension assessments with the use of the technology could demonstrate at least good-to-excellent reliability except for the intra-rater reliability of “flexion lateral”.

## Background

Total knee replacement (TKR) is one of the most frequently performed elective procedures in the field of orthopedic surgery across the globe [1]. The most common indication for the procedure is osteoarthritis, which, as a potentially disabling condition, poses a leading public health burden worldwide [2]. TKR is an effective surgical treatment to improve function and reduce pain when conservative or medical treatment fails [3].

Soft tissue balance, in addition to accurate implant alignment, is one of the major determinants for the success of TKR contributing to the durability of the prostheses and long-term clinical outcomes [4]. Joint instability due to soft tissue imbalance was found to be culpable for one-fourth of revision TKR operations in the literature [5–7].

In conventional TKR, soft tissue balancing is performed by orthopedic surgeons after the bone cut, depending on subjective judgment, with the knee extended and flexed 90 degrees [8]. The introduction of new technologies in recent years, such as intra-articular pressure sensors and robotic systems, has provided orthopedic surgeons with objective quantifiable measurements for evaluating soft tissue balance throughout the entire range of motion [9–11].

One of the commercially-available robotic-assisted TKR systems is CORI (Smith and Nephew Inc., USA) [12]. Ligament tension assessment is an important step in the surgical procedure of robotic-assisted TKR. The information it generated is essential for the planning of bone cut (i.e., component position), implant sizing, and guiding soft tissue release. Therefore, the reliability of ligament tension assessment is crucial for the system. During the ligament tension assessment, the robotic system measures joint gap size between tibia and femur of the knee joint. Varus and valgus stress are manually applied across the entire range of motion.

As a new field of orthopedic surgery, literature has provided little evidence regarding the inter-rater and intra-rater reliability of CORI in performing gap balancing. A study conducted in Japan looked at the inter-rater reliability of the CORI ligament tension assessment and suggested that the result might be affected by the experience of the surgeon. The experienced surgeon produced a larger gap in both the medial and lateral compartments at nearly all flexion angles [13].

The objective of this study was to determine the inter-rater and intra-rater reliability of performing gap balancing in the CORI robotic-assisted TKR in the present study. We hypothesized that CORI robotic-assisted TKR ligament tension assessment has a high inter-rater and intra-rater reliability. Only after the reliability of the system is established could we further proceed to investigate the optimal figures for soft tissue balance, as well as to proceed with further investigations to determine if there are long-term clinical benefits for our patients.

## Methods

### Overview of design

This was a prospective study conducted from 19 January 2022 to 24 February 2023. The inclusion criteria were patients over 40 years old who suffered from end-stage osteoarthritis of the knee (Kellgren and Lawrence grade 3–4) and underwent CORI robotic-assisted TKR. The implant used was a Journey II BCS (Smith and Nephew Inc., USA). The exclusion criteria included post-traumatic arthritis, active infection or sepsis, revision surgery, medial or lateral collateral ligament insufficiency, significant knee deformities (varus deformity > 30 or valgus deformity > 20) and not meeting the indications for TKR according to the specific Smith & Nephew Knee System’s Instructions For Use (IFU). Ethical approval was obtained from the Institutional Ethics Review Committee (Ref. No.: CRE 2022.146).

Clinical data collected included age, gender, body mass index (BMI), preoperative maximum extension, and flexion angles of the patients. The data from the ligament tension assessment during CORI TKR planning stage were collected. The values for the medial and lateral compartment gap during flexion and extension were recorded. Patients’ demographics, knee range of motion, and BMI were also recorded.

### Surgical procedures and gap quantification

**Fig. 1:**
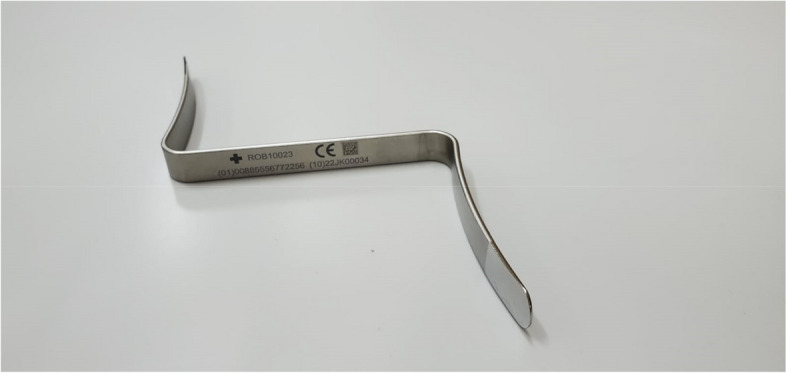
Z-shaped special retractor for distraction of medial and lateral joint gap

Three orthopedic specialists with extensive experience in CORI robotic-assisted TKR (having performed at least 30 robotic-assisted TKR surgeries in the preceding year) served as the chief surgeons, and six orthopedic trainees who were inexperienced in CORI TKR system worked as assistants in the study. During the operation, osteophyte removal was performed first. During ligament tension assessment, a Z-shaped special retractor (Fig. 1) was inserted into the medial and lateral joint space to help tension the respective collateral ligament. Gap assessment was performed in the following orders: (1) the chief surgeon applied varus force to tension the lateral collateral ligament throughout the range of motion, followed by valgus force applied to tension the medial collateral ligament. The ligament tension assessment data were saved and labeled as S1; (2) the assistant surgeon repeated the procedure, and the ligament tension assessment data were saved and labelled as A1; (3) the chief surgeon repeated the process with varus and valgus force again. The ligament tension assessment data were saved and labeled as S2. Each step (S1, A1, S2) in the ligament tension assessment was repeated three times and the CORI robot recorded the largest joint gap size attained in all three trials. The surgeons were instructed to apply varus and valgus force until firm endpoint was felt. Only the extension gap and flexion gap of the medial and lateral compartments generated by CORI robots were used for comparison instead of the whole range of motion data to simulate ligament tension assessment as in conventional TKR.

### Statistical analysis

Statistical analysis was performed using IBM SPSS Statistics for Windows, Version 26.0. (IBM Corp, Armonk, NY, USA). For the intra-rater reliability, intra-class correlation (ICC) estimates and their 95% confidence intervals (95% CI) were calculated based on a mean rating (*k* = 2), absolute agreement, and 2-way mixed-effects model. For the inter-rater reliability, a two-way random effects model was used. Cohen’s Kappa values with 95% CI were calculated to look for inter-rater and intra-rater agreements. An ICC of 1 indicated perfect reliability. An ICC of < 0.5 was indicative of poor reliability, values between 0.5 and 0.75 indicated moderate reliability, values between 0.75 and 0.9 indicated good reliability, and values greater than 0.9 indicated excellent reliability [14]. A Pearson correlation was computed to determine the relationship between joint gap size and preoperative Hip-knee-ankle (HKA) angles. If the coefficient value lay between ± 0.50 and ± 1.00, it indicated a strong correlation. If the value stood between ± 0.30 and ± 0.49, it indicated a medium correlation. When the value was below ± 0.29, it indicated a weak correlation.

## Results

**Fig. 2:**
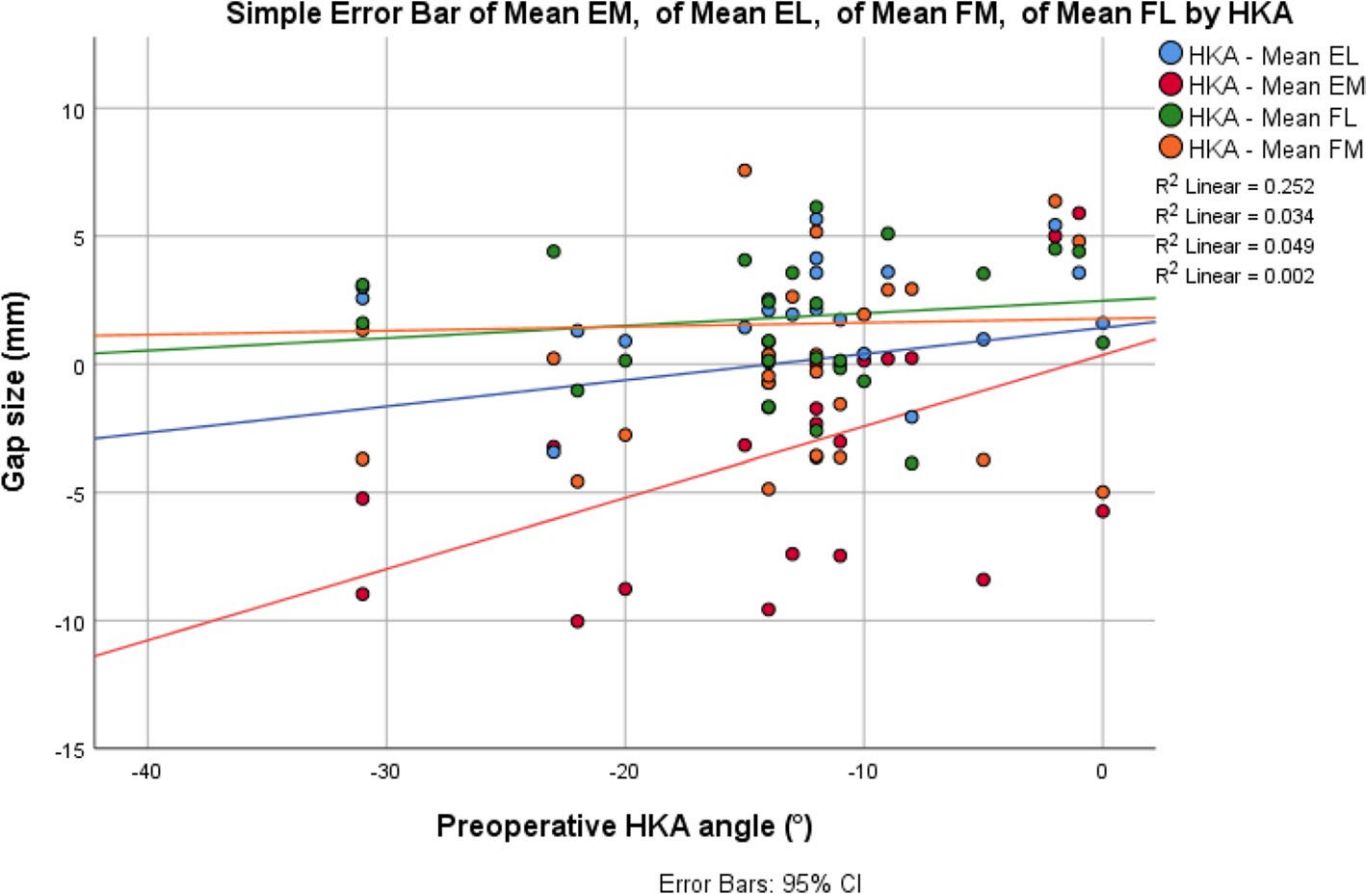
Scatter plot showing joint gap size (means of chief surgeon assessments and assistant surgeon assessments) and preoperative Hip-knee-ankle (HKA) angle (EM: extension medial; EL: extension lateral; FM: flexion medial; FL: flexion lateral)

Twenty-four knees in 21 patients with end-stage osteoarthritis who underwent CORI robotic-assisted TKR were recruited. The demographics of the patients are summarized in Table 1. The mean age of the patients at operation was 74.8 years (SD): 13.7). Among the 24 knees, 19 were female patients and 5 were male ones. Eight knees were from patients with BMI > 30. The mean knee range of motion was 98.1 (SD: 15.1). All patients had either varus or neutrally-aligned knee osteoarthritis. The mean joint gap size of extension medial (EM), extension lateral (EL), flexion medial (FM), and flexion lateral (FL) was − 2.97 mm ± 4.23 mm, 1.83 mm ± 2.08 mm, 0.07 mm ± 3.68 mm, 1.57 mm ± 2.61 mm, respectively. Figure 2 shows the scatter plot of joint gap size and preoperative HKA angle. EM gap size and the preoperative HKA were found to be strongly correlated, [r(22) = 0.50, *P* = 0.01]. In addition, there was also a non-significant relationship between preoperative HKA angles and EL [r(22) = 0.19, *P* = 0.39], FM [r(22) = 0.22, *P* = 0.30], and FL [r(22) = 0.05, *P* = 0.83].

**Table 1:** Demographics of patients (*n* = 24)

Age (years), Mean ± SD	71.8 ± 6.0
Gender	Female:Male = 19:5
BMI (kg/m^2^), Mean ± SD	28.1 ± 4.5
Preoperative maximum extension angle (°), Mean ± SD	8.1 ± 8.4
Preoperative maximum flexion angle (°), Mean ± SD	106.3 ± 9.2
Knee Range of Motion (°), Mean ± SD	98.1 ± 15.1
Pre-operative hip knee ankle angle (°), Mean ± SD	− 13.2 ± 7.8

*SD* Standard deviation, *BMI* Body mass index

**Fig. 3:**
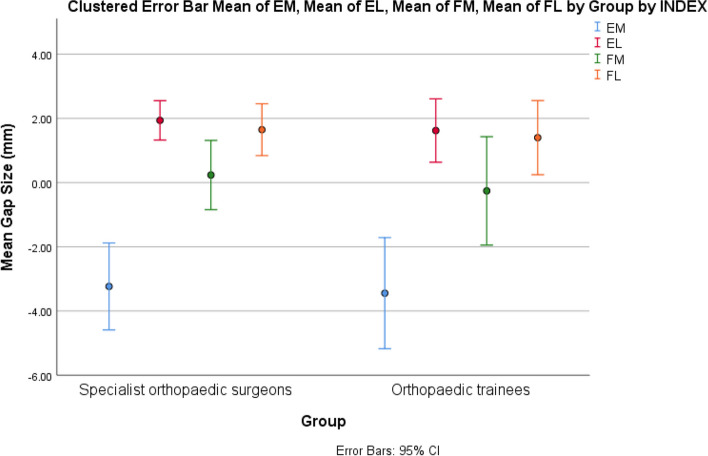
Means and 95% CI of joint gap size for extension medial (EM), extension lateral (EL), flexion medial (FM), flexion lateral (FL) grouped by specialist orthopedic surgeons vs. orthopedic trainees (CI: Confidence interval)

Figure 3 shows the mean and 95% CI of joint gap sizes of EM, EL, FM, and FL grouped according to surgeon experience (specialist orthopaedic surgeons versus orthopaedic trainees). No significant differences exist between the two groups was demonstrated.

Inter-rater reliability in joint gap assessment was assessed by comparing S1/A1/S2 data (Table 2). Flexion lateral had the worst reliability and extension medial had the best reliability. In terms of ICC (95% CI), the medial gap in both extension and flexion had excellent reliability whilst the lateral gap in extension and flexion had good-to-excellent reliability.

**Table 2:** Inter-rater reliability between surgeons and assistants

S1-A1-S2	ICC (95% CI)	Cronbach’s α
Extension Medial	0.972 (0.944–0.987)	0.971
Extension Lateral	0.938 (0.879–0.971)	0.941
Flexion Medial	0.956 (0.913–0.980)	0.959
Flexion Lateral	0.933 (0.868–0.969)	0.931

S1: The chief surgeon applied varus force to tension the lateral collateral ligament throughout the range of motion, followed by valgus force to tension the medial collateral ligament. The ligament tension assessment data were saved and labelled as S1

A1: The assistant surgeon repeated the procedure, and the ligament tension assessment data were saved and labelled as A1

S2: The chief surgeon repeated the process with varus and valgus force applied again. The ligament tension assessment data were saved and labelled as S2

ICC: Intra-class correlation; CI: Confidence interval

Table 3 shows the intra-rater reliability of joint gap assessment between 2 measurements by the chief surgeon. Similar to inter-rater reliability, flexion lateral had the worst reliability, and extension medial had the best reliability. Extension medial showed excellent reliability, extension lateral and flexion medial showed good-to-excellent reliability, and flexion lateral showed moderate-to-excellent reliability.

**Table 3:** Intra-rater reliability of between 2 measurements by the chief surgeons

S1-S2	ICC (95% CI)	Cronbach’s α
Extension Medial	0.967 (0.923–0.986)	0.965
Extension Lateral	0.928 (0.833–0.969)	0.931
Flexion Medial	0.905 (0.781–0.959)	0.909
Flexion Lateral	0.885 (0.732–0.950)	0.881

S1: The chief surgeon applied varus force to tension the lateral collateral ligament throughout the range of motion, followed by valgus force to tension the medial collateral ligament. The ligament tension assessment data were saved and labelled as S1

S2: The chief surgeon repeated the process with varus and valgus force applied again. The ligament tension assessment data were saved and labelled as S2

ICC: Intra-class correlation; CI: Confidence interval

## Discussion

Accurate assessment of joint gap size is essential for achieving favorable outcomes in TKR, as it helps decide the optimal position and size of the prosthetic components, ensuring proper balance and stability of the joint and improved postoperative outcomes, including Knee Society Knee Score, Western Ontario and McMaster Universities Osteoarthritis Index, Forgotten Joint Score [15, 16]. Conventionally, ligament tension assessment is done manually [8, 17]. However, manual method is not quantitative, is often subject to inter-rater variability, and relies heavily on the surgeon's experience [8, 17, 18]. The recent technologies, such as intra-articular pressure sensors, have provided orthopedic surgeons with objective quantifiable measurements for evaluating soft tissue balance [10, 11]. However, these electronic sensors are used after bone resection. Surgeons must perform tibia and femur cut before quantitative data on ligament tension can be reviewed.

The robotic-assisted technology has emerged as a promising solution to address these challenges, in the hope for more precise and reproducible joint gap assessment. In 2022, Sohmiya et al. published research on the reliability of joint gap size assessment using the CORI surgical system [13]. They showed that there existed an inter-rater difference in joint distraction force with the senior surgeon having a larger joint gap size compared with the junior surgeon. In another cadaveric study, 7 arthroplasty surgeons examined the inter-rater and intra-rater reliability of the ligament tension assessment using the CORI surgical system [19]. There was inconsistency in measured joint gap sizes collected during the range of motion both among surgeons (inter-rater variability) and for each surgeon (intra-rater variability). The use of a Z retractor during gap tensioning helped to provide more mechanical leverage and to stress the ligaments in deep flexion. This trend was only observed on the lateral side though, and the author postulated this might be related to the looser nature of the lateral compartment, which led to a higher variability in measured joint gap sizes. A recent study evaluated the repeatability and reproducibility of ligamentous laxity assessment of robotic-assisted total knee replacement using a digital tensioner in 12 cadaveric knees with three experienced surgeons [20]. On average, for pre-resection ligamentous laxity assessment, the variation within a surgeon was 0.33 ± 0.26 mm and 0.69 ± 0.33 mm when compared among different surgeons. These results were consistent with our findings of excellent repeatability and reproducibility achieved in ligamentous laxity assessment in robotic-assisted total knee replacement.

This study looked into the reliability of ligament tension assessment with CORI surgical system. We showed that, for both inter-rater and intra-rater reliability, extension and medial joint gap assessment had better reliability whilst flexion and lateral joint gap assessment yielded worse reliability. However, all ligament tension assessments had at least good-to-excellent reliability except flexion lateral for intra-rater assessment. For this study, we involved 3 specialist orthopedic surgeons and 6 orthopaedic trainees so that the data produced were more generalizable. This lays a platform for future research on the performance of balancing assessments in robotic-assisted total knee replacement, and the impact of different alignment strategies on patients’ functional outcomes.

## Conclusions

In conclusion, robotic-assisted technology provides a reliable solution for improving ligament tension assessment. Flexion and lateral joint gap size assessment are associated with worse reliability.

## Data Availability

The datasets used and/or analysed during the current study are available from the corresponding author on reasonable request.

## Abbreviations

TKR: Total knee replacement
IFU: Instructions for Use
BMI: Body Mass Index
ICC: Intra-class correlation
CI: Confidence interval
HKA: Angle Hip-knee-ankle angle
SD: Standard deviation
EM: Extension medial
EL: Extension lateral
FM: Flexion medial
FL: Flexion lateral
